# Effect of metformin on hypoxia-associated gene expression in oral cavity squamous cell carcinoma in non-diabetic patients - a prospective window of opportunity study

**DOI:** 10.1007/s00405-025-09493-8

**Published:** 2025-06-04

**Authors:** Simon A. Mueller, Olgun Elicin, Bastien Monney, Tilman Rau, Alan Dal Pra, Ludwig Sachs, Irene Centeno Ramos, Erik Vassella, Florian Dammann, Francesca Caparrotti, Andreas Limacher, Lluís Nisa, Matthias S. Dettmer, Roland Giger

**Affiliations:** 1 Department of Oto-Rhino-Laryngology, Head and Neck Surgery, Inselspital, Bern University Hospital, University of Bern, Bern, Switzerland; 2 Department of Oto-Rhino-Laryngology, Head and Neck Surgery, University Hospital Zurich, Zurich, Switzerland; 3 Department of Radiation Oncology, Inselspital, Bern University Hospital and University of Bern, Bern, Switzerland; 4 Department for BioMedical Research, University of Bern, Bern, Switzerland; 5 Institute of Tissue Medicine and Pathology, University of Bern, Bern, Switzerland; 6 Institute of Pathology, University Clinic Duesseldorf, Duesseldorf, Germany; 7 Department of Radiation Oncology, University of Miami Health System, Coral Gables, FL USA; 8 Interventional and Pediatric Radiology, University Institute of Diagnostic, Inselspital, Bern University Hospital, University of Bern, Bern, Switzerland; 9 Générale-Beaulieu Swiss Oncology Network, Geneva, Switzerland; 10 Department of Clinical Research, CTU Bern, University of Bern, Bern, Switzerland; 11 Institute of Pathology, Klinikum Stuttgart Katharinenhospital, Stuttgart, Germany

**Keywords:** Oral squamous cell carcinoma, Tumor hypoxia, Metformin, Radiation-Sensitizing agents, Gene expression profiling, Prospective studies

## Abstract

**Purpose:**

Research suggests that metformin may reduce tumor hypoxia, rendering it a potential radiosensitizer. This study investigated metformin’s effect on hypoxia-associated gene expression in oral cavity squamous cell carcinoma (OCSCC).

**Methods:**

In this prospective trial, non-diabetic patients with OCSCC scheduled for curative surgery received preoperative metformin for 10 to 14 days. Tumor biopsies were taken before and after metformin treatment. Gene expression profiling was performed using RNASeq, emphasizing on hypoxia-associated signaling pathways.

**Results:**

Fifteen patients of 25 enrolled patients completed the study protocol and passed quality checks. No significant difference in gene expression was observed after adjustment for multiple testing. No significant changes were found in the hypoxia-associated hypoxia-inducible factor 1- alpha (HIF1A) pathway or in other signaling pathways associated with metformin reported in the literature.

**Conclusion:**

This study does not support the hypothesis that metformin improves tumor hypoxia in OCSCC after 10 to 14 days of treatment.

**Supplementary Information:**

The online version contains supplementary material available at 10.1007/s00405-025-09493-8.

## Introduction

Primary surgery and risk-adapted adjuvant radio(chemo)therapy (RCT) is the preferred treatment for advanced oral cavity squamous cell carcinoma (OCSCC) with an estimated 5-year overall survival of 50% [1]. In mucosal head and neck squamous cell carcinomas (HNSCC) in other locations - the pharynx and larynx - as well as in selected cases of OCSCC, RCT may be applied as the primary treatment. Resistance to radiation is therefore an important prognostic risk factor. A major cause of radioresistance is tumor hypoxia [2], and researchers have focused their interest on finding new approaches to address this problem.

Metformin (1,1-dimethylbiguanide hydrochloride) is an oral drug that belongs to the biguanide class of hypoglycemic agents. Approved in 1958, it has been the drug of choice for the treatment of type 2 diabetes, although its exact mechanism of action is still not fully understood [3]. Recently, the drug has gained renewed attention due to its potentially positive effects in oncology. Epidemiological evidence suggests that taking metformin lowers the risk of cancer and reduces cancer deaths in diabetics [4, 5]. In addition, a systematic review and meta-analysis demonstrated that metformin improves the prognosis of HNSCC [6]. Whether this beneficial effect also applies to non-diabetic patients is unknown. There are several hypotheses on how metformin inhibits tumor growth, mostly through inhibition of respiratory complex I in the mitochondria and the activation of AMPK in succession [7]. Animal models suggest that inhibition of the mammalian target of rapamycin (mTOR) may play a role [7, 8]. Metformin may also favorably alter the metabolic and immunological tumor microenvironment in HNSCC patients, by reducing hypoxia and increasing the CD8/FOXP3 ratio, respectively [9, 10, 11, 12]. Preclinical models suggest that metformin could serve as a radiosensitizer by reducing tissue hypoxia [9, 13]. To test this hypothesis in a clinical setting for HNSCC, we conducted a prospective, single-arm study using metformin before surgery in non-diabetic patients with OCSCC to determine changes in hypoxia associated gene expression.

## Materials and methods

This single-center, single-arm, open-label window-of-opportunity study was conducted at Inselspital, Bern University Hospital, Bern, Switzerland, a tertiary referral center. It was approved by the Bern Ethics Committee (ID 2017–02276). All participants provided written consent.

### Patients and study procedures

Non-diabetic patients with biopsy-proven OCSCC, age > 18 years, were eligible. Exclusion criteria were non-surgical treatment of the tumor or distant metastasis, pregnancy or lactation, prior metformin treatment, any condition prohibiting the use of metformin or any condition potentially causing tissue hypoxia and/or increased risk of lactic acidosis.

**Fig. 1:**
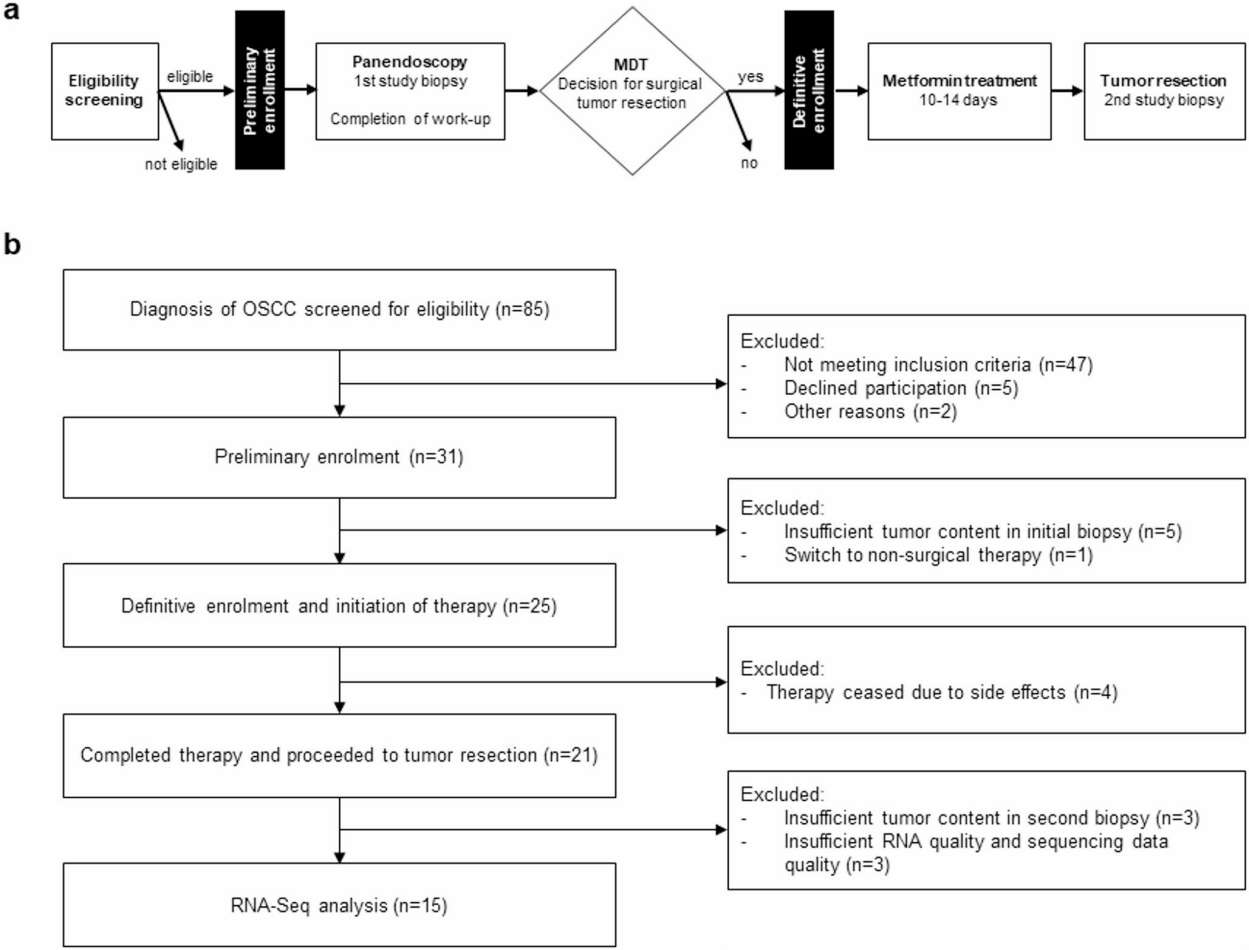
**(a)** Study flowchart and **(b)** CONSORT flow chart. MDT, multidisciplinary tumor board; OCSCC, oral cavity squamous cell carcinoma

The study design is shown in Fig. 1a. During a routine diagnostic panendoscopy under general anesthesia, a tumor biopsy was taken and processed according to a specifically designed protocol detailed in the subsequent section. Tumor work-up was completed according to institutional standards. If the multidisciplinary tumor board (MDT) recommended surgical resection of the primary tumor, the participants were definitely enrolled. Before the surgery, patients were given 850 mg metformin twice a day for a minimum of 10 to a maximum of 14 days, with the last dose administered the evening before surgery. The individual duration of treatment within the predefined range of 10–14 days depended on the per protocol acceptable delay between MDT and the planned surgery. Patients recorded each administered dose and leftover medication was collected before surgery to assess patients’ compliance. At the beginning of the surgery, the second biopsy was taken using the identical protocol as during panendoscopy.

A gadolinium contrast-enhanced magnetic resonance imaging (MRI) of the head and neck was obtained as part of the diagnostic work-up in all patients recruited for the study. A second MRI was performed on the day before surgery in patients who had completed their metformin treatment using the identical imaging protocol. Tumor size, relative signal intensity change after gadolinium administration, apparent diffusion co-efficient (ADC) and diffusion-coefficient were compared (Figure S1).

### Sample collection

To avoid bias introduced by oxygen depletion after disconnection of the tissue samples from blood supply, we established a strict pipeline to keep cold ischemia time to an absolute minimum (< 30 s) and avoid any thawing of the samples before RNA extraction. Herein, samples were snap-frozen in the operating theater. Immediately after biopsy, the tissue was placed on a paper disc (Biosystems, Muttenz, Switzerland) inside a cryomold embedding dish (Tissue-Tek^®^ Sakura Finetek, Alphen, Netherlands), covered with optimal cutting temperature (OCT) compound (CellPath, Newtown, United Kingdom) and frozen at -20 °C. Isopentane was cooled using dry ice and the freshly removed cryomold was placed here for 5 min and then transported on dry ice to the pathology laboratory.

Frozen section from the OCT block with hematoxylin and eosin (H&E) staining was performed. The OCT blocks were stored at -80 °C at the Tissue Bank Bern. The pathologist on duty defined the tumor content based on the H&E staining. In case tumor content was < 70%, the biopsy was repeated in the same surgery until sufficient tumor content was achieved. If repeat biopsies were insufficient or the tumor too small for additional biopsies, the patient was excluded from the study. From five patients, additional biopsies of adjacent healthy mucosa were processed in identical fashion. These were used to explore if any changes in gene expression were tumor specific or equally present in healthy mucosa.

### RNA extraction

A specialist head and neck pathologist (MSD) defined the area for RNA extraction. Punches were taken from the selected area (1 mm punch with ejector; Kai Medical, Solingen, Germany), for which the OCT block was placed for 10 min at -20 °C inside a cryostat (Leica Biosystems, Wetzlar, Germany). The frozen punch was transferred into a 2 ml tube containing 200 µL of RNA extraction buffer (AM1560, Invitrogen, Waltham, MA, USA) and a stainless steel bead (Qiagen, Hilden, Germany). Tissue homogenization was performed using TissueLyser II (Qiagen), according to the manufacturer’s instructions, using 2 cycles for 2 min at 25 Hz. Total RNA extraction was achieved using the mirVana™ miRNA Isolation Kit, with phenol (ThermoFisher, Waltham, MA, USA) according to the manufacturer’s protocol. Quantity and quality of purified RNA were assessed by Qubit 4.0 fluorometer with the Qubit™ RNA BR Assay Kit (ThermoFisher, Q10211) and Advanced Analytical Fragment Analyzer System (Agilent, Santa Clara, CA, USA, DNF-471).

### RNA sequencing

Sequencing libraries were established using Illumina TruSeq^®^ Stranded mRNA Library Prep kit (Illumina, San Diego, CA, USA, 20020595) in combination with TruSeq^®^ RNA UD Indexes (Illumina, 20022371) according to Illumina’s guidelines. Pooled cDNA libraries were sequenced paired-end using a shared Illumina NovaSeq 6000 S4 Reagent Kit (300 cycles; Illumina, 20028312) on an Illumina NovaSeq 6000 instrument. The run produced a minimum of 50 million reads/sample. Quality of the sequencing run was assessed using Illumina Sequencing Analysis Viewer version 2.4.7 (Illumina) and all base call files were demultiplexed and converted into FASTQ files using Illumina bcl2fastq conversion software version 2.20. The quality control assessments, generation of libraries and sequencing were conducted by the Next Generation Sequencing Platform, University of Bern.

### Bioinformatic processing

Quality control and preprocessing of sequencing data were performed using fastp (v0.19.5) [14], with reads with a Phred score below 20 and a minimal length of 50 being filtered. HISAT2 (v2.1.0) [15] was used to align sequencing reads to the reference human genome assembly GRCh38. Alignment data were processed with SAMtools (v1.8) [16] and quantification of annotated reads was assessed with featurecounts (v2.0.1) [17]. Reference genome assembly datasets were obtained from the Ensembl release 106 [18].

Statistical analysis of differential gene expression was conducted by non-parametric Wilcoxon signed rank test on each identified genomic feature. Additional analyses were performed using DESeq2 (v1.38.3) [19] and EdgeR (v.3.40.2) [20, 21] to corroborate initial findings. A multiple-test corrected p-value of 0.05 (q-value) was used in all three methods to assess the false discovery rate (FDR) [22]. Differentially expressed genes (DEG) at the uncorrected significance level of 0.05, i.e. before applying the FDR adjustment, were subsequently used for Generally Applicable Gene-set Enrichment (GAGE) [23] and REACTOME Pathway Analysis [24] to identify genetic pathways enriched for DEG.

## Results

Thirty-one non-diabetic patients were recruited (Fig. 1b). Six were excluded because of low tumor content in the first biopsy (*n* = 5) and switch to non-surgical treatment (*n* = 1). Twelve adverse events were recorded in the 25 patients undergoing metformin treatment, which included diarrhea (*n* = 7), obstipation (*n* = 1), dyspnea (*n* = 1), tumor-associated dysphagia (*n* = 1; increased pain (*n* = 1), and wound infection (*n* = 1), leading to discontinuation of treatment in four patients (dyspnea, *n* = 1; dysphagia, *n* = 1; diarrhea, *n* = 2). All adverse events resolved after discontinuation of metformin.

Of the 21 patients who completed the course of metformin, six were excluded due to insufficient tumor content in the second biopsy (*n* = 3) and insufficient quality of extracted RNA (*n* = 3), leaving 15 patients for the final analysis. Median duration of metformin intake in the final cohort was 12 days (range 10–14 days). Mean compliance with the metformin prescription (consumed number of the totally prescribed number of pills) was 98% (range 86–100%).

In 16 of the 21 patients who completed the course of metformin, a second MRI was performed the day before surgery (this cohort contained 11 patients that underwent RNASeq and 5 where RNASeq was not possible, Table S1). Median time between the MRIs was 26 days (range 13–40 days). While the mean tumor size increased by 34% (SD 57%, *p* = 0.05), no significant differences in contrast-agent intensity, ADC and diffusion coefficient where detected (Figure S1).

An overview of the clinical and pathologic data of the 15 patients included in the RNASeq analysis is presented in Table 1. All included patients were white Europeans. The cohort shows high prevalence of smoking and alcohol intake with an age distribution typical of OCSCC. Seven patients underwent surgery only, while eight patients underwent adjuvant radiotherapy (*n* = 4, 27%) or chemo-radiotherapy (*n* = 4, 27%). Eight patients (53%) remained free of tumor, while five (33%) suffered recurrence (local, *n* = 2; regional, *n* = 1; loco-regional, *n* = 2) and four (27%) developed second primary carcinomas (larynx, *n* = 2; hard palate, *n* = 1; pancreas, *n* = 1). Mean follow up was 47.4 months (range 6.9 to 76.7). At last follow-up, 10 patients (67%) were alive and disease free, two (13%) succumbed to OCSCC, while three (20%) died of other causes. Of patients receiving radiotherapy (*n* = 4, 27%) or chemo-radiotherapy (*n* = 4, 27%), five (33%) were disease free at last follow-up (one patient died of OSCC, 2 died of other causes).

**Table 1:** Clinical and pathologic characteristics of the cohort undergoing RNA-sequencing

Characteristic	Cohort (*n* = 15)
Age in years, median (range)	65.6 (59.8 to 76.9)
Sex, n (%)	
female	6 (40)
male	9 (60)
Smoking, n (%)	
non-smoker	3 (20)
active	9 (60)
ceased	3 (20)
Smoking pack years, n (%)	
non-smoker (< 1 pack year)	3 (20)
≥1 to < 30 pack years	2 (13)
≥30 pack years	10 (67)
Alcohol, n (%)	
none	2 (13)
ceased	2 (13)
active, 1–2 units/day^a^	9 (60)
active, > 2 units/day^a^	2 (13)
Tumor site, n (%)	
tongue	4 (27)
floor of mouth	6 (40)
hard palate	1 (7)
alveolar crest	3 (20)
buccal mucosa	1 (7)
Days of metformin treatment, median (range)	12 (10–14)
Compliance to prescribed metformin intake, mean percentage (range)	97.8 (86.4–100)
pT-Stage, n (%)	
T1	4 (27)
T2	6 (40)
T3	2 (13)
T4a	3 (20)
pN-Stage^b^, n (%)	
N0	8 (53)
N1	2 (13)
N2a/b/c or N3a	0
N3b	5 (33)
AJCC/UICC 8th edition [25], n (%)	
Stage I	4 (27)
Stage II	3 (20)
Stage III	2 (13)
Stage IVA	6 (40)
p16 - Status	
negative	15 (100)
positive	0 (0)
Type of surgical resection, n (%)	
wide excision	2 (13)
wide excision, unilateral neck dissection	6 (40)
wide excision, bilateral neck dissection	7 (47)
Surgical reconstruction, n (%)	
none	4 (27)
pedicled flap	1 (7)
free flap	10 (67)
Resection status, n (%)	
R0	14 (93)
R1 (no ink on tumor but close margin < 1 mm)	1 (7)
Lympho-vascular infiltration, n (%)	
yes	6 (40)
no	9 (60)
Perineural invasion, n (%)	
yes	6 (40)
no	9 (60)
Grading	
G1	1 (7)
G2	13 (87)
G3	1 (7)
Extracapsular spread in lymph node metastasis, n (%)	
NA (pN0)	8 (53)
no	2 (13)
yes	5 (33)
Adjuvant therapy	
none	7 (47)
Radiotherapy	4 (27)
Chemo-radiotherapy	4 (27)

^a^Units defined as 3 dl beer = 1 dl wine = 0.2 dl of spirits. ^b^For the two patients where neck dissection was not performed, cN stage is listed instead of pN stage

### Differential gene expression analysis

**Fig. 2:**
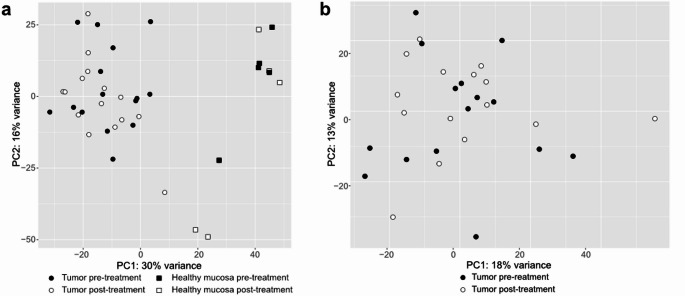
Principal component analysis (PCA) of the total gene expression before and after treatment with metformin. **(a)** PCA of 15 OCSCC tumor and 5 normal mucosal controls. While tumors and controls are clearly distinguishable, samples before and after treatment of both groups do not separate. **(b)** PCA of only tumor samples shows no separation between samples before and after metformin treatment

Quality assessment showed an overall excellent quality of the sequencing data, with an average quality score (Phred [26]) across all bases > 30 for all included samples and a very low adapter content before trimming. A total number of 734 significant DEG were identified between pre-treatment and post-treatment tumor samples with an uncorrected p-value of < 0.05. When corrected for multiple testing and considering a q-value of 0.05 (and equally at 0.1), none of these genes retained statistical significance. In principal component analysis, tumor samples were readily distinguished from healthy mucosa, but a clear distinction between the samples before and after treatment was found neither in tumors nor in healthy mucosa (Fig. 2).

### Pathway analysis

**Fig. 3:**
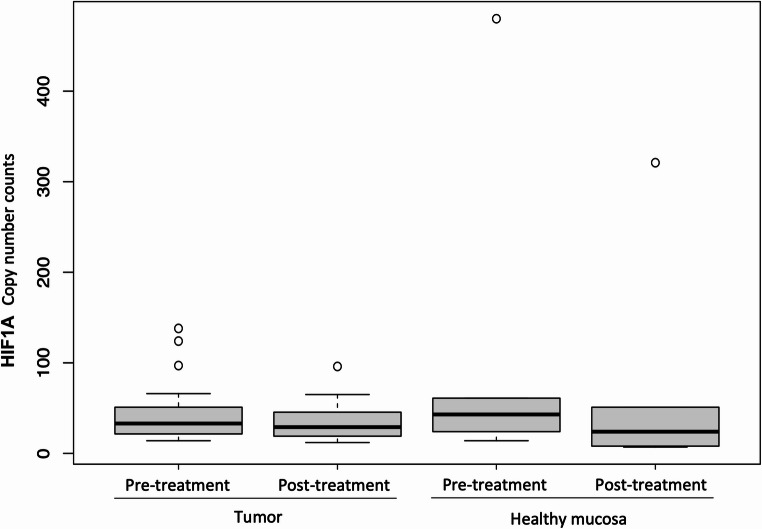
Expression of HIF1A in tumor and mucosal samples pre and post treatment with metformin

The primary endpoint of the study was the activity of the hypoxia-inducible factor 1 subunit alpha (HIF1A) and its associated pathway before and after treatment. The expression of HIF1A, the main regulator of the hypoxia response, did not differ significantly between tumor and normal samples obtained before and after treatment with metformin (Fig. 3).

**Fig. 4:**
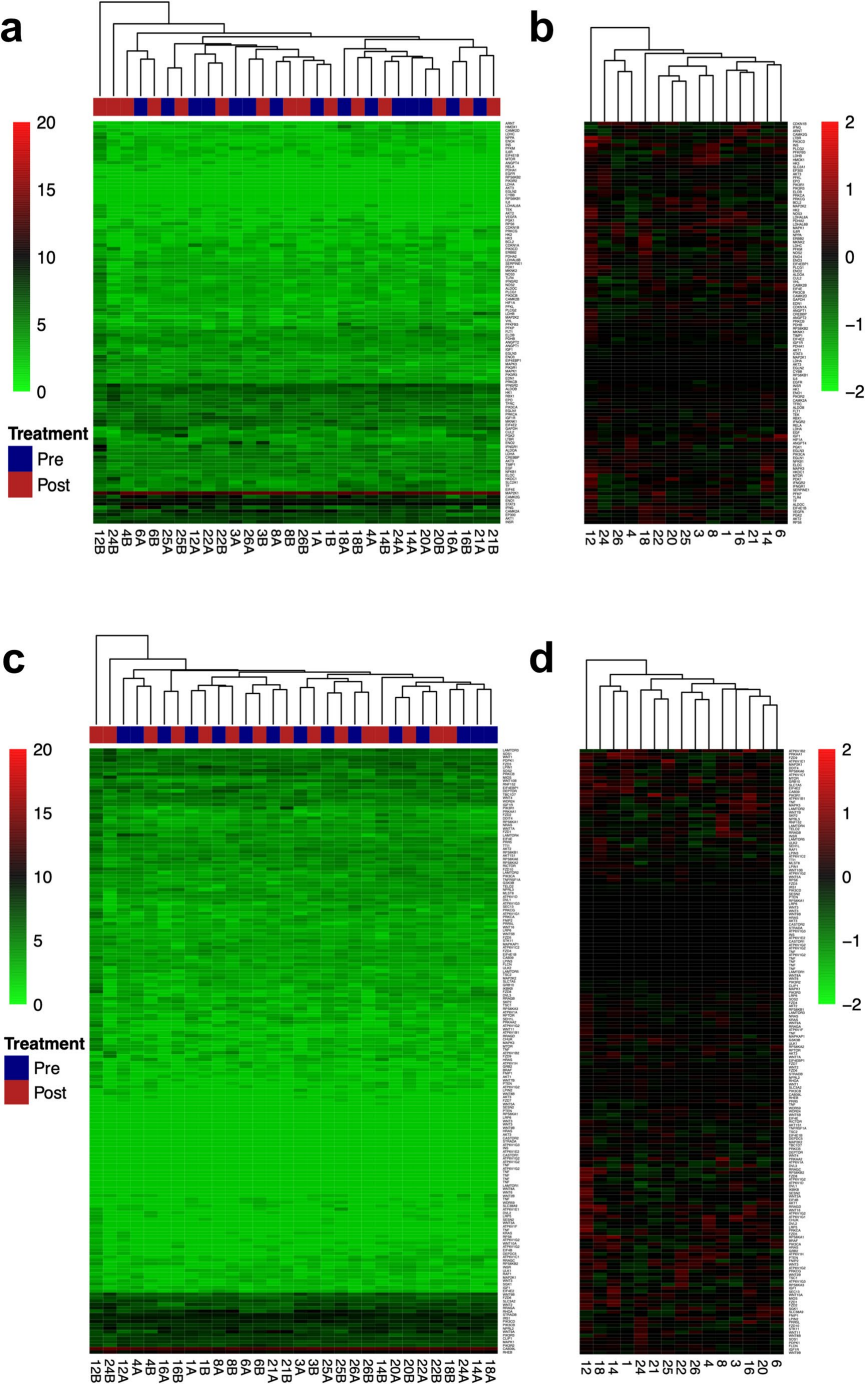
Pathway analysis of OCSCC before and after metformin treatment. **(a)** Expression of genes attributed to the HIF1A-pathway. Heatmap depicting raw expression values of genes (red and green scale). The dendrogram on top of the heatmap illustrates the clustering of samples, which does not separate samples pre and post metformin treatment; **(b)** Heatmap illustrating the difference in expression of the individual genes of HIF1A-pathway in each of the 15 cancer patients. Scale on the right shows fold-change of gene expression. None of the genes showed a consistent change in expression across the cohort. **(c)** Expression of genes of the mTOR-pathway in OCSCC before and after metformin treatment. Heatmap depicting the raw expression values of genes. The dendrogram above the heatmap illustrates the clustering of samples where the samples before and after metformin treatment are not separated. **(d)** Heatmap illustrating the differences in expression of the individual genes of the mTOR-pathway in each of the 15 cancer patients. None of the genes showed a consistent change in expression across the cohort

Pathway analysis, which included all genes associated with the HIF1A pathway according to the Kyoto Encyclopedia of Genes and Genomes (KEGG) [ 27] revealed no significant changes in pathway expression in tumor and before and after treatment with metformin (Fig. 4a and b).

Likewise, no significantly DEG within the mTOR pathway before and after metformin treatment (Fig. 4c and d) was revealed, nor in any other pathway.

## Discussion

In this prospective window-of-opportunity study, we tested the hypothesis that metformin would lead to measurable changes in hypoxia-associated gene expression and reduce the activity of the HIF1A signaling pathway. To the best of our knowledge, no previous studies in OCSCC or any other tumor type have been published, in which the transcriptome was analyzed holistically using RNASeq before and after metformin treatment. Surprisingly, we did not detect any change in gene expression of hypoxia-associated genes or any other significant change in gene expression.

A pivotal study conducted almost 20 years ago found that metformin use was associated with a lower risk of cancer in patients with type 2 diabetes, suggesting that there may be a dose-response relationship [28]. Epidemiological studies suggest that HNSCC with type 2 diabetes who take metformin have a lower cancer-related mortality rate [29, 30].

Preclinical studies found that one of the underlying mechanisms of action of metformin in OCSCC is the inhibition of mTOR and c-MYC expression [31]. Moreover, although metformin inhibits cell growth and induces apoptosis of cultured OCSCC cells in a dose-dependent manner through p-AMPK activation, these effects are abrogated in the presence of normal stromal cells [32], suggesting that the effect of metformin on OCSCC cells may be modulated by the microenvironment. The intriguing hypothesis and inspiration for this study is that metformin reduces tumor hypoxia, which in turn is influenced by the microenvironment, and is associated with radioresistance [9, 33].

### Metformin and tissue hypoxia

Hypoxia plays a crucial role in tumorigenesis, including angiogenesis [34] and impairs response to radiation [33]. HIF1A, a heterodimeric transcription factor, is sensitive to cellular oxygen levels. Hypoxic conditions lead to its overexpression and the activation of target genes such as vascular endothelial growth factor (VEGF) [34]. Targeting the expression of HIF1A is therefore seen as a potential strategy for cancer therapy. Several studies demonstrated that metformin is able to inhibit the HIF1A signaling pathway in vitro, leading to increased cell death in OCSCC and gallbladder carcinoma [35, 36]. Metformin was shown to inhibit oxygen consumption of cancer cells in vitro and in vivo, reducing tumor hypoxia and significantly enhancing the radiation response of xenografts [9]. In a randomized phase II trial of RCT in stage IB–IVA cervical cancer metformin reduced tumor hypoxia in selected patients with hypoxic tumors, as measured by fluoroazomycin arabinoside positron emission tomography. This translated into a significant improvement of 2-year disease-free survival [37]. Using photoacoustic imaging, metformin at high doses was also shown to increase oxygenation in HNSCC xenografts in mice after five days [38]. A dose finding study of metformin in combination with concurrent cisplatin and radiotherapy in patients with locally advanced HNSCC showed promising results [39].

We reasoned that these promising results should reflect in hypoxia-associated RNA expression. Surprisingly, we detected no significant changes in the expression of HIF1A or other genes of the HIF1A pathway in both tumor and normal tissue samples collected from our patients before and after metformin intake. Given the rigor of our tissue processing pipeline and the high quality of the sequencing data, our results allow to rule out strong effects of metformin and major changes in expression despite the limited sample size. We cannot exclude the possibility that a longer course or higher dose of metformin would reveal a significant effect. Indeed, one recent animal study found that metformin’s immunomodulatory effects appeared only after long-term metformin treatment, including prior to tumor inoculation [12]. Longer treatment was impossible in our study setting, as it would have caused an unacceptable delay of treatment. Meanwhile, a higher dose would have increased the rate of gastrointestinal side effects. Nevertheless, several published studies on breast and endometrial cancer using similar or lower dosing and only slightly longer treatment demonstrated an effect of metformin in pre- and post-therapeutic biopsies in immunohistochemistry or RNA microarray [40, 41, 42], although the focus of these studies was not on hypoxia or the HIF1A pathway. The aforementioned study by Han et al. [37] demonstrated a reduction in tumor hypoxia in cervical carcinoma, but unlike OCSCC, cervical cancer is typically associated with human papilloma virus (HPV) [43]. Thus, it represents a different tumor not only in location, but also in pathophysiology and tumor microenvironment [44].

### Metformin and the mTOR-pathway

In vitro studies report that the intracellular chain of events triggered by metformin begins in the mitochondria by reducing the energy charge in the cell, leading to the activation of AMP-activated protein kinase (AMPK). Its activation leads to inhibition of the mTOR pathway, reduction in proliferation, reduction in mRNA translation and protein synthesis in cancer cells in vitro [45, 46]. mTOR inhibition disturbs protein synthesis, resulting in a potent antineoplastic effect mediated by the inhibition of the proto-oncogene c-MYC and HIF1A [47]. Not least because of this interaction with HIF1A, we specifically investigated the mTOR pathway, but again did not detect any significant difference in the expression of its genes after metformin treatment.

The absence of a short-term effect of metformin may be specific to OCSCC. On the other hand, Gutkind et al. demonstrated that a course of metformin over 12 weeks reduced the proliferation rate and mTOR-signaling in premalignant lesions of the oral mucosa [48]. More than half of their participants had a partial or complete histological response of their lesion. Since the study assessed oral premalignant lesions rather than OCSCC and the analysis consisted of immunohistochemistry rather than RNASeq, we cannot draw any inference with regard to our primary study endpoints. Nevertheless, the results by Gutkind et al. suggest that measurable alterations of gene expression may ensue after a longer course of metformin [48]. In OCSCC, such long neoadjuvant treatment is not ethically justifiable given the insufficient evidence that its potential effects would warrant a delay of treatment. Of note, three phase II trials investigating metformin’s effect on premalignant oral lesions are currently ongoing (clinicaltrials.gov identifier NCT05237960, NCT02581137, NCT05727761).

### Potentials and limitations of metformin use in HNSCC patients

While our results do not support the hypothesis of a metformin-associated reduction in tumor hypoxia, studies assessing the tumor microenvironment have shown an effect after a similar duration and dosage of metformin in HNSCC. Notably, an increase of cytotoxic CD8 + T-lymphocytes, decrease of regulatory FOXP3 + lymphocytes and increased function of natural killer cells in tumors has been described, supporting the hypothesis that metformin may favorably alter the tumor microenvironment [9, 10, 11, 12, 39, 49]. A clinical trial combining metformin with the immune checkpoint inhibitor durvalumab in operable HNSCC (ClinicalTrial identifier NCT03618654) found a trend for early pathologic response that did not reach significance level [50]. The results of another trial combining metformin and pembrolizumab in metastatic HNSCC (NCT04414540) are still pending.

Our study highlights a potential limitation of the use of metformin in non-diabetic OCSCC patients. Over one third of the originally enrolled participants (38%) reported diarrhea following metformin intake, which led to discontinuation in two cases (8%). Hadad et al. reported similar difficulties in their breast cancer cohort [40]. A slow dose build-up may mitigate gastrointestinal side effects. In HNSCC, the toxicity of RCT may further restrict alimentation and limit the application of concomitant metformin. This problem was highlighted in two recent studies combining RCT with metformin in HNSCC patients. The first phase I/II trial was discontinued because of adverse events in 8 of 18 (44%) patients [39]. The second, a phase I/II study equally highlighted that metformin’s gastrointestinal toxicity makes its widespread use challenging during RCT [51]. In this study, the oncologic outcome of patients with compliance of metformin intake > 70% did not improve significantly compared to those whose compliance was < 70%, a fact speaking against metformin’s potential as a radiosensitizer. We are not aware of any other currently ongoing trial investigating metformin in combination with radiotherapy for HNSCC.

### Limitations

Apart from the above mentioned confinements in dosage and treatment duration, the limitations of our study are its relatively small sample size, which was in part due to a temporary halt of recruitment during the COVID-19 pandemic. Given the excellent quality of the sequencing data, our results nevertheless allow to rule out strong effects of metformin. The subinvestigation comparing pre- and post-treatment MRI did not include the identical cohort that underwent RNASeq, thus the results of both analyses may not be correlated. Lastly, potential immunomodulatory effects of metformin were not assessed via systematically collected blood samples at the time of biopsy and surgery, which may be a subject of interest in future research.

## Conclusion

This comprehensive analysis comparing the transcriptome of 15 OCSCC before and after metformin treatment revealed no significant changes in gene expression and, in particular, no changes in the expression of hypoxia-associated signaling pathways. Although we cannot exclude the possibility that a longer treatment could eventually lead to detectable changes in the expression of hypoxia-associated genes, our study does not support the hypothesis that metformin improves tumor hypoxia in OCSCC.

## Electronic supplementary material

Below is the link to the electronic supplementary material.

Supplementary Material 1

## References

[1] Cooper JS, Pajak TF, Forastiere AA et al (2004) Postoperative concurrent radiotherapy and chemotherapy for high-risk squamous-cell carcinoma of the head and neck. N Engl J Med 350(19):1937–1944. 10.1056/NEJMoa032646 15128893 10.1056/NEJMoa032646

[2] Gray LH, Conger AD, Ebert M, Hornsey S, Scott OC (1953) The concentration of oxygen dissolved in tissues at the time of irradiation as a factor in radiotherapy. Br J Radiol 26(312):638–648. 10.1259/0007-1285-26-312-638 13106296 10.1259/0007-1285-26-312-638

[3] Hundal RS, Krssak M, Dufour S et al (2000) Mechanism by which Metformin reduces glucose production in type 2 diabetes. Diabetes 49:2063–2069. 10.2337/diabetes.49.12.2063 11118008 10.2337/diabetes.49.12.2063 PMC2995498

[4] Pollak MN (2012) Investigating Metformin for Cancer prevention and treatment: the end of the beginning. Cancer Discov 2:778–790. 10.1158/2159-8290.CD-12-0263 22926251 10.1158/2159-8290.CD-12-0263

[5] Hatoum D, McGowan EM (2015) Recent advances in the use of metformin: can treating diabetes prevent breast cancer? BioMed Res Int 2015:548436. 10.1155/2015/548436 25866793 10.1155/2015/548436 PMC4383151

[6] Jiao Y, Liu D, Sun Y, Chen Z, Liu S (2022) Survival benefit of Metformin as an adjuvant treatment for head and neck cancer: A systematic review and Meta-Analysis. Front Pharmacol 13:850750. 10.3389/fphar.2022.850750 35645803 10.3389/fphar.2022.850750 PMC9136048

[7] Hua Y, Zheng Y, Yao Y, Jia R, Ge S, Zhuang A (2023) Metformin and cancer hallmarks: shedding new lights on therapeutic repurposing. *Journal of Translational Medicine*. /06/21 2023;21(1):403. 10.1186/s12967-023-04263-8 10.1186/s12967-023-04263-8 PMC10286395 37344841

[8] Chae YK, Arya A, Malecek M-K et al (2016) Repurposing Metformin for cancer treatment: current clinical studies. Oncotarget 7:40767–40780. 10.18632/oncotarget.8194 27004404 10.18632/oncotarget.8194 PMC5130043

[9] Zannella VE, Dal Pra A, Muaddi H et al (2013) Reprogramming metabolism with Metformin improves tumor oxygenation and radiotherapy response. Clin Cancer Res 19:6741–6750. 10.1158/1078-0432.CCR-13-1787 24141625 10.1158/1078-0432.CCR-13-1787

[10] Scharping NE, Menk AV, Whetstone RD, Zeng X, Delgoffe GM (2017) Efficacy of PD-1 Blockade is potentiated by Metformin-Induced reduction of tumor hypoxia. Cancer Immunol Res 5(1):9–16. 10.1158/2326-6066.Cir-16-0103 27941003 10.1158/2326-6066.CIR-16-0103 PMC5340074

[11] Amin D, Richa T, Mollaee M et al (2020) Metformin effects on FOXP3 + and CD8 + T cell infiltrates of head and neck squamous cell carcinoma. Laryngoscope 130(9):E490–E498. 10.1002/lary.28336 31593308 10.1002/lary.28336

[12] Veeramachaneni R, Yu W, Newton JM et al (2021) Metformin generates profound alterations in systemic and tumor immunity with associated antitumor effects. J Immunother Cancer 9(7):e002773. 10.1136/jitc-2021-002773 34230113 10.1136/jitc-2021-002773 PMC8261884

[13] De Bruycker S, Vangestel C, Staelens S et al (2019) Effects of Metformin on tumor hypoxia and radiotherapy efficacy: a [(18)F]HX4 PET imaging study in colorectal cancer xenografts. EJNMMI Res Aug 2(1):74. 10.1186/s13550-019-0543-4 10.1186/s13550-019-0543-4 PMC6677842 31375940

[14] Chen S, Zhou Y, Chen Y, Gu J (2018) Fastp: an ultra-fast all-in-one FASTQ preprocessor. Bioinformatics 34(17):i884–i890. 10.1093/bioinformatics/bty560 30423086 10.1093/bioinformatics/bty560 PMC6129281

[15] Kim D, Paggi JM, Park C, Bennett C, Salzberg SL (2019) Graph-based genome alignment and genotyping with HISAT2 and HISAT-genotype. Nat Biotechnol 37(8):907–915. 10.1038/s41587-019-0201-4 31375807 10.1038/s41587-019-0201-4 PMC7605509

[16] Danecek P, Bonfield JK, Liddle J et al (2021) Twelve years of samtools and BCFtools. GigaScience 10(2). 10.1093/gigascience/giab008 10.1093/gigascience/giab008 PMC7931819 33590861

[17] Liao Y, Smyth GK, Shi W (2013) FeatureCounts: an efficient general purpose program for assigning sequence reads to genomic features. Bioinformatics 30(7):923–930. 10.1093/bioinformatics/btt656 24227677 10.1093/bioinformatics/btt656

[18] Cunningham F, Allen JE, Allen J et al (2021) Ensembl 2022. Nucleic Acids Res 50(D1):D988–D995. 10.1093/nar/gkab1049 10.1093/nar/gkab1049 PMC8728283 34791404

[19] Love MI, Huber W, Anders S (2014) Moderated Estimation of fold change and dispersion for RNA-seq data with DESeq2. Genome Biol 15:550. 10.1186/s13059-014-0550-8 25516281 10.1186/s13059-014-0550-8 PMC4302049

[20] Robinson MD, McCarthy DJ, Smyth GK (2010) EdgeR: a bioconductor package for differential expression analysis of digital gene expression data. Bioinformatics 26(1):139–140. 10.1093/bioinformatics/btp616 19910308 10.1093/bioinformatics/btp616 PMC2796818

[21] Chen Y, Lun AT, Smyth GK (2016) From reads to genes to pathways: differential expression analysis of RNA-Seq experiments using Rsubread and the edger quasi-likelihood pipeline. F1000Res 5:1438. 10.12688/f1000research.8987.2 27508061 10.12688/f1000research.8987.1 PMC4934518

[22] Benjamini Y, Hochberg Y (1995) Controlling the false discovery rate: A practical and powerful approach to multiple testing. J R Stat Soc Ser B Stat Methodol 57(1):289–300. 10.1111/j.2517-6161.1995.tb02031.x

[23] Luo W, Friedman MS, Shedden K, Hankenson KD, Woolf PJ (2009) GAGE: generally applicable gene set enrichment for pathway analysis. BMC Bioinformatics 10(1):161. 10.1186/1471-2105-10-161 19473525 10.1186/1471-2105-10-161 PMC2696452

[24] Yu G, He Q-Y (2016) ReactomePA: an R/Bioconductor package for reactome pathway analysis and visualization. Mol Biosyst 12(2):477–479. 10.1039/C5MB00663E 26661513 10.1039/c5mb00663e

[25] Brierley JG, Mary K, Wittekind C (2017) TNM classification of malignant tumours, 8 edn. Wiley-Blackwell

[26] Ewing B, Hillier L, Wendl MC, Green P (1998) Base-calling of automated sequencer traces using phred. I. Accuracy assessment. Genome Res 8(3):175–185. 10.1101/gr.8.3.175 9521921 10.1101/gr.8.3.175

[27] Kanehisa M, Furumichi M, Sato Y, Kawashima M, Ishiguro-Watanabe M (2023) KEGG for taxonomy-based analysis of pathways and genomes. Nucleic Acids Res 51(D1):D587–d592. 10.1093/nar/gkac963 36300620 10.1093/nar/gkac963 PMC9825424

[28] Evans JM, Donnelly LA, Emslie-Smith AM, Alessi DR, Morris AD (2005) Metformin and reduced risk of cancer in diabetic patients. BMJ 330(7503):1304–1305. 10.1136/bmj.38415.708634.F7 15849206 10.1136/bmj.38415.708634.F7 PMC558205

[29] Stokes WA, Eguchi M, Amini A et al (2018) Survival impact and toxicity of Metformin in head and neck cancer: an analysis of the SEER-Medicare dataset. Oral Oncol 84:12–19. 10.1016/j.oraloncology.2018.06.022 30115470 10.1016/j.oraloncology.2018.06.022 PMC6659721

[30] Ogunsakin A, Infield J, Zuber J, Solomon SS (2018) Metformin associated with improved outcomes in diabetic patients with laryngeal and oropharyngeal carcinoma. Am J Med Sci 356(6):574–575. 10.1016/j.amjms.2018.09.002 30342717 10.1016/j.amjms.2018.09.002 PMC7673203

[31] Wang Y, Zhang Y, Feng X et al (2021) Metformin inhibits mTOR and c-Myc by decreasing YAP protein expression in OSCC cells. Oncol Rep 45(3):1249–1260. 10.3892/or.2020.7909 33650651 10.3892/or.2020.7909

[32] Zhang Z, Liang X, Fan Y et al (2019) Fibroblasts rescue oral squamous cancer cell from metformin-induced apoptosis via alleviating metabolic disbalance and inhibiting AMPK pathway. Cell Cycle 18(9):949–962. 10.1080/15384101.2019.1598727 31014173 10.1080/15384101.2019.1598727 PMC6527302

[33] Wouters BG, Weppler SA, Koritzinsky M et al (2002) Hypoxia as a target for combined modality treatments. Eur J Cancer 38:240–257. 10.1016/S0959-8049(01)00361-6 11803141 10.1016/s0959-8049(01)00361-6

[34] Ren Y, Luo H, Metformin (2021) The next angiogenesis panacea? SAGE Open Med 9:20503121211001641. 10.1177/20503121211001641 33796300 10.1177/20503121211001641 PMC7970164

[35] Guimarães TA, Farias LC, Santos ES et al (2016) Metformin increases PDH and suppresses HIF-1α under hypoxic conditions and induces cell death in oral squamous cell carcinoma. Oncotarget 7(34):55057–55068. 10.18632/oncotarget.10842 27474170 10.18632/oncotarget.10842 PMC5342401

[36] Ye J, Chen K, Qi L et al (2018) Metformin suppresses hypoxia–induced migration via the HIF–1α/VEGF pathway in gallbladder cancer in vitro and in vivo. Oncol Rep 40(6):3501–3510. 10.3892/or.2018.6751 30272364 10.3892/or.2018.6751

[37] Han K, Fyles A, Shek T et al (2022) A phase II randomized trial of chemoradiation with or without Metformin in locally advanced cervical Cancer. Clin Cancer Res 28(24):5263–5271. 10.1158/1078-0432.Ccr-22-1665 36037303 10.1158/1078-0432.CCR-22-1665

[38] Verma A, Rich LJ, Vincent-Chong VK, Seshadri M (2018) Visualizing the effects of Metformin on tumor growth, vascularity, and metabolism in head and neck cancer. J Oral Pathol Med 47(5):484–491. 10.1111/jop.12705 29573032 10.1111/jop.12705 PMC5966030

[39] Gulati S, Desai J, Palackdharry SM et al (2020) Phase 1 dose-finding study of Metformin in combination with concurrent cisplatin and radiotherapy in patients with locally advanced head and neck squamous cell cancer. Cancer 126(2):354–362. 10.1002/cncr.32539 31626727 10.1002/cncr.32539 PMC10402880

[40] Hadad S, Iwamoto T, Jordan L et al (2011) Evidence for biological effects of Metformin in operable breast cancer: a pre-operative, window-of-opportunity, randomized trial. Breast Cancer Res Treat 128(3):783–794. 10.1007/s10549-011-1612-1 21655990 10.1007/s10549-011-1612-1

[41] Dowling RJ, Niraula S, Chang MC et al (2015) Changes in insulin receptor signaling underlie neoadjuvant Metformin administration in breast cancer: a prospective window of opportunity neoadjuvant study. Breast Cancer Res 17(1):32. 10.1186/s13058-015-0540-0 25849721 10.1186/s13058-015-0540-0 PMC4381495

[42] Schuler KM, Rambally BS, DiFurio MJ et al (2015) Antiproliferative and metabolic effects of Metformin in a preoperative window clinical trial for endometrial cancer. Cancer Med 4(2):161–173. 10.1002/cam4.353 25417601 10.1002/cam4.353 PMC4329001

[43] Nauta IH, Heideman DAM, Brink A et al (2021) The unveiled reality of human papillomavirus as risk factor for oral cavity squamous cell carcinoma. Int J Cancer 149(2):420–430. 10.1002/ijc.33514 33634865 10.1002/ijc.33514 PMC8251537

[44] Beaty BT, Cosper PF, Beriwal S, Weiner AA (2021) Why De-Intensification is not possible in HPV-Associated cervical Cancer. Semin Radiat Oncol 31(4):339–348. 10.1016/j.semradonc.2021.06.001 34455989 10.1016/j.semradonc.2021.06.001

[45] Pernicova I, Korbonits M (2014) Metformin–mode of action and clinical implications for diabetes and cancer. Nat Rev Endocrinol 10(3):143–156. 10.1038/nrendo.2013.256 24393785 10.1038/nrendo.2013.256

[46] Amin S, Lux A, O’Callaghan F (2019) The journey of Metformin from glycaemic control to mTOR Inhibition and the suppression of tumour growth. Br J Clin Pharmacol 85(1):37–46. 10.1111/bcp.13780 30290005 10.1111/bcp.13780 PMC6303203

[47] Blandino G, Valerio M, Cioce M et al (2012) Metformin elicits anticancer effects through the sequential modulation of DICER and c-MYC. Nat Commun 3:865. 10.1038/ncomms1859 22643892 10.1038/ncomms1859

[48] Gutkind JS, Molinolo AA, Wu X et al (2021) Inhibition of mTOR signaling and clinical activity of Metformin in oral premalignant lesions. JCI Insight Sep 8(17):e147096. 10.1172/jci.insight.147096 10.1172/jci.insight.147096 PMC8492350 34255745

[49] Crist M, Yaniv B, Palackdharry S et al (2022) Metformin increases natural killer cell functions in head and neck squamous cell carcinoma through CXCL1 Inhibition. J Immunother Cancer 10(11):e005632. 10.1136/jitc-2022-005632 36328378 10.1136/jitc-2022-005632 PMC9639146

[50] Philips R, Alnemri A, Tekumalla S et al (2023) 629-B Window of opportunity for durvalumab plus Metformin trial in head & neck squamous cell carcinoma (HNSCC). J Immunother Cancer 11(Suppl 2):A1807–A1807. 10.1136/jitc-2023-SITC2023.0629-B

[51] Kemnade JO, Florez M, Sabichi A et al (2023) Phase I / II trial of Metformin as a chemo-radiosensitizer in a head and neck cancer patient population. Oral Oncol 145:106536 2023/10/01. 10.1016/j.oraloncology.2023.106536 37562095 10.1016/j.oraloncology.2023.106536

