# Supplementary material for: Effect of metformin on hypoxia-associated gene expression in oral cavity squamous cell carcinoma in non-diabetic patients - a prospective window of opportunity study
Source: Eur Arch Otorhinolaryngol. 2025 Jun 4;282(9):4773–84. doi: 10.1007/s00405-025-09493-8 (PMC12423204; doi:10.1007/s00405-025-09493-8)

**Effect of Metformin on Hypoxia-Associated Gene Expression in Oral Cavity Squamous Cell Carcinoma in Non-Diabetic Patients - a Window of Opportunity Study**

**Supplement**

**Journal:** European Archives of Oto-Rhino-Laryngology

**Authors**

Simon A. Mueller^1,2^, Olgun Elicin^3^, Bastien Monney^1,4^, Tilman Rau^5,6^ , Alan Dal Pra^7^, Ludwig Sachs^1^, Irene Centeno Ramos^7^, Erik Vassella^7^, Florian Dammann^8^, Francesca Caparrotti^9^ , Andreas Limacher^10^, Lluís Nisa^1^, Matthias S. Dettmer^5,11^, Roland Giger^1^

Corresponding Author:

Simon A. Mueller simon.mueller@usz.ch

**Affiliations**

1. Department of Oto-Rhino-Laryngology, Head and Neck Surgery, Inselspital, Bern University Hospital and University of Bern, Bern, Switzerland
2. Department of Oto-Rhino-Laryngology, Head and Neck Surgery, University Hospital Zurich, Zurich, Switzerland
3. Department of Radiation Oncology, Inselspital, Bern University Hospital and University of Bern, Bern, Switzerland
4. Department for BioMedical Research, University of Bern, Bern, Switzerland
5. Institute of Tissue Medicine and Pathology, University of Bern, Bern, Switzerland
6. Institute of Pathology, University Clinic Duesseldorf, Duesseldorf, Germany
7. Department of Radiation Oncology, University of Miami Health System, Coral Gables, Florida, USA
8. University Institute of Diagnostic, Interventional and Pediatric Radiology, Inselspital, Bern University Hospital and University of Bern, Bern, Switzerland
9. Générale-Beaulieu Swiss Oncology Network, Geneva, Switzerland
10. CTU Bern, Department of Clinical Research, University of Bern, Bern, Switzerland
11. Institute of Pathology, Klinikum Stuttgart Katharinenhospital, Stuttgart, Germany

**Table S1.** Clinical and pathologic characteristics of the cohort undergoing MRI pre and post metformin treatment.

| Characteristic | Cohort (n=16) |
| --- | --- |
| Age in years, median (range) | 64.3 (53.0 to 75.5) |
| Sex, n (%) | |
| female | 6 (38) |
| male | 10 (63) |
| Smoking, n (%) | |
| non-smoker | 3 (19) |
| active | 10 (63) |
| ceased | 3 (19) |
| Smoking pack years, n (%) |  |
| non-smoker (<1 pack year) | 3 (19) |
| ≥1 to <30 pack years | 2 (13) |
| ≥30 pack years | 11 (69) |
| Alcohol, n (%) | |
| none | 3 (19) |
| ceased | 2 (13) |
| active, 1-2 units/day^a^ | 9 (56) |
| active, >2 units/day^a^ | 2 (13) |
| Tumor site, n (%) | |
| tongue | 4 (25) |
| floor of mouth | 6 (38) |
| hard palate | 2 (13) |
| alveolar crest | 2 (13) |
| buccal mucosa | 2 (13) |
| Days of metformin treatment, median (range) | 12 (10 - 14) |
| Compliance to prescribed metformin intake, mean percentage (range) | 96.5 (86.4 - 100) |
| pT-Stage, n (%) | |
| T1 | 5 (27) |
| T2 | 6 (38) |
| T3 | 2 (13) |
| T4a | 3 (19) |
| pN-Stage^b^, n (%) | |
| N0 | 9 (56) |
| N1 | 1 (6) |
| N2a/b/c or N3a | 0 |
| N3b | 6 (38) |
| AJCC/UICC 8^th^ edition [21], n (%) | |
| Stage I | 4 (25) |
| Stage II | 4 (25) |
| Stage III | 1 (6) |
| Stage IVA | 7 (44) |
| p16 - Status |  |
| negative | 15 (94) |
| Positive | 1 (6) ^c^ |
| Type of surgical resection, n (%) | |
| wide excision | 3 (19) |
| wide excision, unilateral neck dissection | 5 (31) |
| wide excision, bilateral neck dissection | 8 (50) |
| Surgical reconstruction, n (%) | |
| none | 3 (19) |
| pedicled flap | 1 (6) |
| free flap | 12 (75) |
| Resection status, n (%) | |
| R0 | 15 (94) |
| R1 (no ink on tumor but close margin <1 mm) | 1 (6) |
| Lympho-vascular infiltration, n (%) | |
| yes | 5 (31) |
| no | 11 (69) |
| Perineural invasion, n (%) | |
| yes | 7 (44) |
| no | 9 (56) |
| Grading | |
| G1 | 3 (19) |
| G2 | 11 (69) |
| G3 | 2 (13) |
| Extracapsular spread in lymph node metastasis, n (%) | |
| NA (pN0 or cN0 without neck dissection) | 9 (56) |
| no | 1 (6) |
| yes | 6 (38) |
| Adjuvant therapy |  |
| none | 8 (50) |
| Radiotherapy | 3 (19) |
| Chemo-radiotherapy | 5 (31) |

^a^Units defined as 3 dl beer = 1 dl wine = 0.2 dl of spirits. ^b^For the patient where neck dissection was not performed, cN stage is listed instead of pN stage. ^c^One patient had intermediate positivity for p16, and HPV was ruled out by PCR.

**Figure S1**. Pre- and post-metformin MRI features. Pre- and post-metformin MRI were available in 16 patients. Mean time between MRI scans was 22 days (SD 9).

a) Tumor size measured in MRI before and after metformin treatment. Mean increase in tumor size was 34% (SD 57.7%, p=0.05). The tumor size was calculated using 3 dimensions (height x, width y, depth z) and the formula π*4/3*x/2*y/2*z2; b) Tumor contrast agent (gadolinium) enhancement between pre- and post-metformin administration. Cancer tissue intensity was measured in T1 pre- and post-contrast sequence. The mean contrast agent value changes between pre- and post-metformin MRI amounted to -0.5 % (SD 48%) and were not statistically significant (p=0.967); c) apparent diffusion coefficient (ADC). ADC was measured over a range of b values from 800 to 1000s/mm. In five of sixteen patients, artifacts precluded a meaningful evaluation of ADC. In the remaining eleven samples, the mean difference of the ADC value was 46.5 (SD 542, p=0.782).

Pre-and post-metformin MRI findings were compared using a paired t-test. The statistical analysis was performed in R and Excel (Microsoft, Redmond, WA, USA).

a b


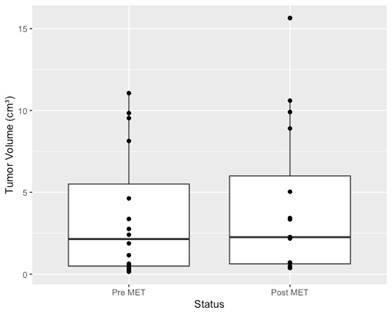

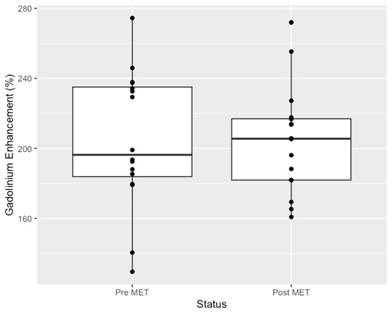


c


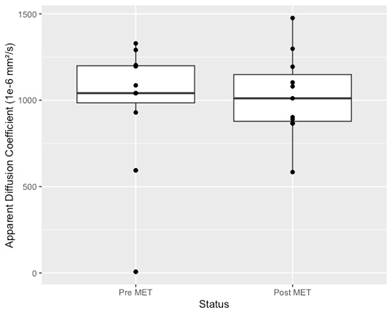

Supplement: Supplementary file 1 — Supplementary Material 1 [file 405_2025_9493_MOESM1_ESM.docx]
